# Ultrastructural Study of *Acanthamoeba polyphaga* Trophozoites and Cysts Treated In Vitro with Cationic Carbosilane Dendrimers

**DOI:** 10.3390/pharmaceutics12060565

**Published:** 2020-06-18

**Authors:** Irene Heredero-Bermejo, Tania Martín-Pérez, José Luis Copa-Patiño, Rafael Gómez, Francisco Javier de la Mata, Juan Soliveri, Jorge Pérez-Serrano

**Affiliations:** 1 Department of Biomedicine and Biotechnology, Faculty of Pharmacy, University of Alcalá, Alcalá de Henares, 28805 Madrid, Spain; tania.martinp@edu.uah.es (T.M.-P.); josel.copa@uah.es (J.L.C.-P.); juan.soliveri@uah.es (J.S.); jorge.perez@uah.es (J.P.-S.); 2 Department of Organic and Inorganic Chemistry, Research Institute on Chemistry “Andrés M. del Río” (IQAR), University of Alcalá, Alcalá de Henares, 28805 Madrid, Spain; rafael.gomez@uah.es (R.G.); javier.delamata@uah.es (F.J.d.l.M.); 3 Institute “Ramón y Cajal” for Health Research (IRYCIS), 28034 Madrid, Spain; 4 Networking Research Center on Bioengineering, Biomaterials and Nanomedicine (CIBER-BBN) ISCIII, 28029 Madrid, Spain

**Keywords:** *Acanthamoeba*, cysts, trophozoites, dendrimers, scanning electron microscopy, transmission electron microscopy

## Abstract

Cationic carbosilane dendrimers are branched molecules with antimicrobial properties. Their activity has been tested against *Acanthamoeba polyphaga*, a causative agent of *Acanthamoeba* keratitis, a severe ocular disease in humans. *A. polyphaga* trophozoites and cysts were exposed to different noncytotoxic cationic carbosilane dendrimers with proven antiamoebic activity. The effects of treatment on cell surface and cell ultrastructure were examined by scanning and transmission electron microscopy, respectively. Two of the dendrimers tested induced dramatic alterations of cellular ultrastructure in both trophozoites and cysts, including vacuolization, depletion of cytoplasmic contents, and reduced cell size. Additionally, we observed severe alterations of the plasma membrane with membrane blebbing in trophozoites and disruption in cysts. These alterations were also observed with chlorhexidine, a drug used for treatment of *Acanthamoeba* keratitis. Our results support that these compounds may target membranes, and their action is critical for parasite integrity.

## 1. Introduction

*Acanthamoeba* spp. are free-living protozoa widely spread in the environment that can cause infections in humans as facultative pathogens. They are associated with a severe eye infection, *Acanthamoeba* keratitis (AK), that usually affects contact lens wearers [1]. Furthermore, the presence of these organisms in water may represent a risk for immunocompromised individuals because of their pathogenicity and ability to act as a vector for viruses and pathogenic bacteria [2]. The number of cases of AK has increased in the last decade, resulting in an estimated incidence of 1 in 21,000 in some areas [3]. The recommended treatment for AK includes a combination of guanidine (0.02% polyhexamethylene biguanide 0.02% chlorhexidine) and diamidine (0.1% propamidine or 0.1% hexamidine) [4]. This therapy approach requires a long treatment that is effective against trophozoites; however, the chronic cyst stage of infection is largely resistant to drug treatment, resulting in persistent infections that often lead to the recurrence of keratitis. In addition to AK, *Acanthamoeba* infection can lead to the development of granulomatous amebic encephalitis (GAE), a rare and fatal disease typically found in immunocompromised individuals [5]. Treatment for GAE is based on multidrug therapy that requires long treatments, generally 6 to 12 months, that are not effective in most cases [6,7]. Therefore, new and more effective therapeutic options are needed for the treatment of these parasitic infections due to the development of drug resistance to current treatments and the inability of these treatments to eliminate cysts.

Many of the current drugs used to treat AK have been found to act on cellular membranes. For example, chlorhexidine, a positively charged molecule, targets the plasma membrane and affects its permeability [8], while polyhexamethylene biguanide (PHMB) interacts nonspecifically with membranes on prokaryotic cells and causes the loss of lipopolysaccharides (LPS) and loss of function in membrane proteins [6]. Furthermore, some diamidines with positive charges interact with amphipathic lipids and produce changes in membrane permeability [9]. Electron microscopic studies of the ultrastructure of trophozoites and cysts have been heavily utilized to investigate the morphological changes after treatment with chlorhexidine diacetate (CHA) and polyhexamethylene biguanide (PHMB) [10], plant compounds [11], chlorine [12], and corifungin [4], among others.

Dendrimers are nanoscopic synthetic molecules that are highly branched with a globular shape, a high density of diverse active surface groups and a monodisperse distribution [13]. They can be used as drugs themselves or as carriers for delivery of other drug molecules. Recently, different studies have provided evidence that various classes of dendrimers present antibacterial, antiviral, antifungal, and antiparasitic activity [14]. Previous work from our laboratory identified some specific classes of dendrimers, such as carbosilane, ammonium, and biguanide dendrimers, that have antiamoebic activity against trophozoites and cysts of *Acanthamoeba* producing growth inhibition and cell death [15,16,17] and antibacterial activity [18,19]. The activity of these compounds depends largely on the multivalency (number of terminal groups) of the molecule; however, the mechanism by which these dendrimers act on *Acanthamoeba* remained unclear. In this study, we used scanning (SEM) and transmission (TEM) electron microscopy approaches to evaluate the morphological and ultrastructural alterations produced by different cationic dendrimers on *A. polyphaga* trophozoites and cysts. We found multiple dendrimers that induced dramatic alterations to the plasma membrane. Importantly, whereas current therapeutics fail to target the cyst stage, the dendrimers in the present study caused dramatic impairment of the membranes of both trophozoites and cysts. These results provide important insight into the mechanism of action by which these dendrimers exert their antiamoebic activities against *Acanthamoeba*.

## 2. Materials and Methods

### 2.1. Acanthamoeba polyphaga

*Acanthamoeba polyphaga* 2961 (a clinical isolate kindly supplied by Dr. E. Hadas, Poznan University of Medical Sciences, Poland) was cultured in peptone–yeast extract–glucose (PYG)–Bactocasitone medium as previously described [20], and incubated at 32 °C.

Cysts were obtained by four days of log-phase culture of trophozoites in nonnutrient Neff’s encystment medium (NEM: 0.1 M KCl, 8 mM MgSO_4_·7H2O, 0.4 mM CaCl_2_·2H_2_O, 1 mM NaHCO_3_, 20 mM ammediol [2-amino-2-methyl-1,3-propanediol; Sigma] pH 8.8 at 32 °C) as previously described [21].

### 2.2. Cationic Carbosilane Dendrimers

**Figure 1:**
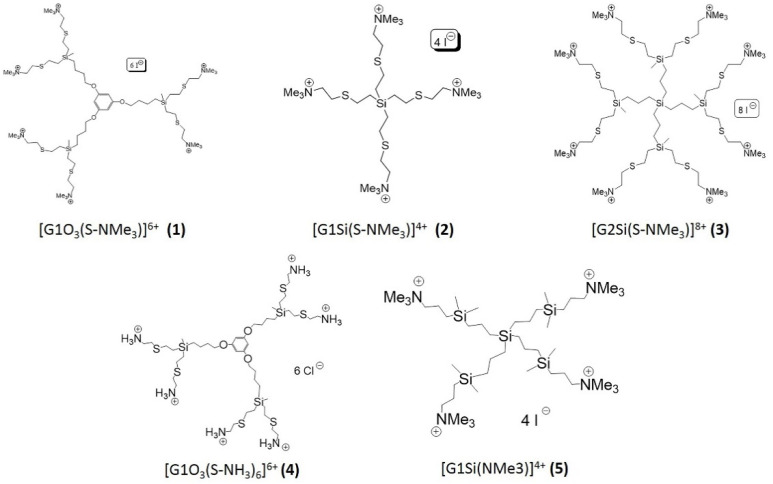
Structures of cationic carbosilane dendrimers used in this work.

All compounds (Table 1) used in this study are carbosilane dendrimers with cationic groups on the periphery, conferring water solubility. The cationic dendrimers tested were [G1O3(S-NMe3)]^6+^ (**1**) (first generation dendrimer with a polyphenoxo core and six terminal trimethylammonium groups), [G1Si(S-NMe3)]^4+^ (**2**) (first generation dendrimer with a silicon atom as core and four terminal trimethylammonium groups), [G2Si(S-NMe3)]^8+^ (**3**) (second generation dendrimer with a silicon atom as core and eight terminal trimethylammonium groups), [G1O3(S-NH3)6]^6+^ (**4**) (first generation dendrimer with a polyphenoxo core and six terminal ammonium groups), [G1Si(NMe3)]^4+^ (**5**) (first generation dendrimer obtained by hydrosilylation with a silicon atom as core and four terminal trimethylammonium groups) (Figure 1) [16,18,19,22]. Their amoebicidal activity and cytotoxicity have been previously described by our group. They were noncytotoxic compounds to cell lines (HeLa and MUPH-1), with high activity and a wide therapeutic window [16,21]. These dendrimers were selected on the basis of their activity as well as for their utility in examining the effects of different core types (a silicon atom or a polyphenoxo moiety), different natures of terminal ammonium groups (NMe^3+^ or NH^3+^), and different numbers of positive charges on their surface. These dendrimers were also obtained using different synthetic procedures, whereas compounds **1**–**4** were obtained through tiol-ene addition reactions and present a sulfur atom in the structure; compound **5** was obtained by hydrosilylation and does not contain this heteroatom, thus changing the hydrophilicity/hydrophobicity balance.

A stock solution of each dendrimer (1024 mg/L) was prepared using distilled sterile water as solvent. Dendrimers were tested at different concentrations against trophozoites and cysts, ranging from 512 to 1 mg/L. Minimum cysticidal concentrations (MCCs) were also tested against cysts. Their half maximal inhibitory concentration (IC_50_) and MCC values are shown in Table 1.

### 2.3. Chlorhexidine Digluconate

Chlorhexidine digluconate (CLX) (Sigma-Aldrich Ltd., St. Louis, MO, USA) was used against *Acanthamoeba* trophozoites and cysts as reference drug (positive control). Concentrations tested ranged from 512 to 1 mg/L. IC_50_ and MCC for CLX were 1.7 ± 0.1 mg/L and MCC, respectively (data obtained from Heredero-Bermejo et al., 2015 [21]).

### 2.4. Scanning Electron Microscopy (SEM)

Trophozoites and cysts were placed in a glass coverslip for 1–2 h and fixed in Milloning’s solution containing 2% glutaraldehyde. Samples were then washed in Milloning’s solution with 0.5% glucose and dehydrated first through an ethanol series and finally with anhydrous acetone. Samples were critical-point dried using a Polaron CPD7501 critical-point drying system, and sputter-coated with 200 Ǻ gold-palladium using a Polaron E5400. Scanning electron microscopy was performed at 5–15 kV in a Zeiss DSM 950 SEM (Carl Zeiss Microscopy GmbH, Jena, Germany).

### 2.5. Transmission Electron Microscopy (TEM)

Amoeba cultures were washed in 0.1 M Milloning’s buffer and fixed in 2% glutaraldehyde solution buffered with 0.1 Milloning’s buffer at pH 7.2 for 2 h (trophozoites) or overnight with slight agitation (cysts). To facilitate thin section preparation, fixed protozoa were embedded in 2% agar. Agarized pellets were then fixed in 1% osmium tetroxide, dehydrated in a graded acetone series, and embedded in Spurr’s resin. Ultramicrotome sections were stained with 1% uranyl acetate followed by 2.5% lead citrate and examined on a Zeiss EM10 TEM (Carl Zeiss Microscopy GmbH, Jena, Germany) at 60 kV.

### 2.6. Statistical Analysis

GraphPad Prism 8 (GraphPad Software, San Diego, CA, USA) was used for to perform Student’s T-tests. Statistical significance indicated as *p*-value less than 0.05.

## 3. Results

### 3.1. Analysis of Trophozoites Morphology

**Figure 2:**
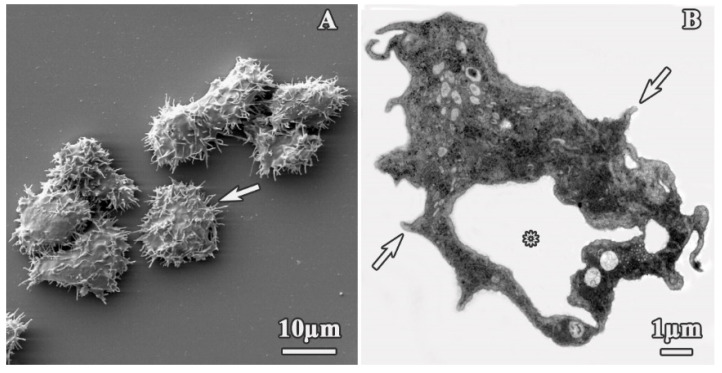
Untreated trophozoites of *A. polyphaga*. (**A**) Scanning electron microscopy (SEM) micrograph and (**B**) transmission electron microscopy (TEM) microphotograph. White arrows: Acanthopodia, asterisk: vacuole.

SEM and TEM imaging were performed to assess structural and ultrastructural alterations induced by cationic dendrimers on *A. polyphaga* 2961 trophozoites. Representative images were selected at concentrations corresponding to approximate IC_50_ values to allow us to observe normal and affected trophozoites and determine the effects of these compounds. For comparison, the morphological structure of untreated trophozoites is shown in Figure 2. These cells exhibited a normal ameboid shape with numerous acanthopodia on the surface (Figure 2A,B, arrows); a size of 20.44 ± 3.1 µm; and a prominent, well-defined vacuole (Figure 2B, asterisk).

**Figure 3:**
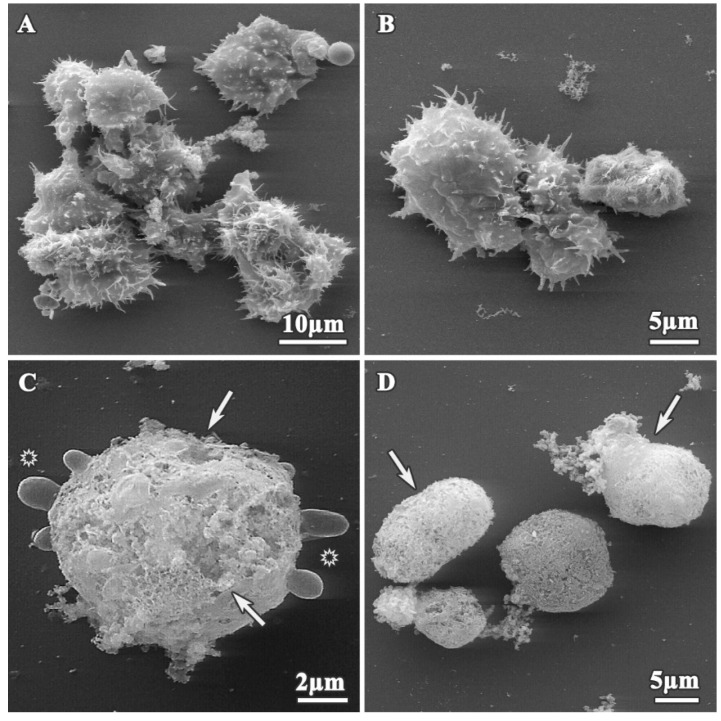
Alterations produced on *A. polyphaga* trophozoites during treatment with dendrimer **1**. Scanning electron microscopy (SEM) micrographs. (**A**,**B**) 16 mg/L, 24 h treatment; (**C**) 64 mg/L, 48 h treatment; (**D**) 256 mg/L, 72 h treatment. White arrows: plasmatic membrane alterations. Asterisk: cell blebbing.

**Figure 4:**
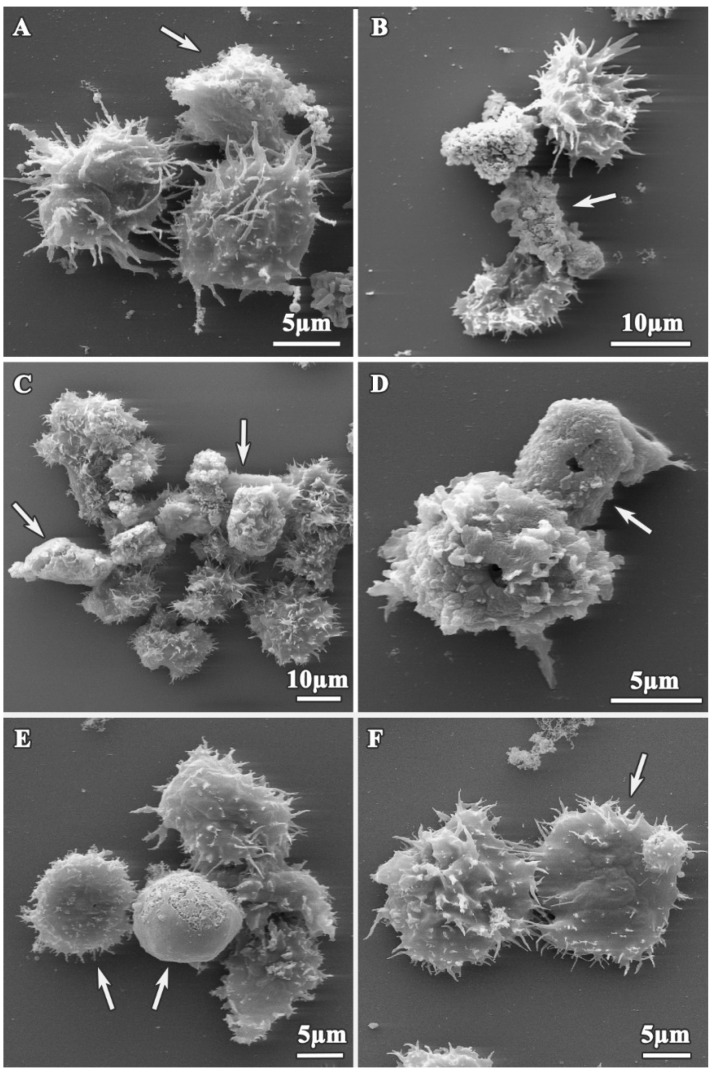
SEM micrograph of *A. polyphaga* trophozoites treated with dendrimers for 24 h. (**A**) 512 mg/L dendrimer **2**; (**B**) 64 mg/L dendrimer **3**; (**C**,**D**) 2 mg/L dendrimer **4**; (**E**,**F**) 8 mg/L dendrimer **5**. Arrow: Plasma membrane alterations.

In contrast, the morphology of the dendrimer-treated trophozoites was altered depending on concentrations and incubation times, as shown on Figure 3 and Figure 4. Following treatment, trophozoites become rounded (Figure 3D and Figure 4C–E) and reduced in size, compared to untreated trophozoites (13.38 ± 1.57 µm, *p* < 0.05) (Figure 3B,D and Figure 4C,E), and showed a reduction in the number of acanthopodia (Figure 3A,B and Figure 4A,C,E,F). The plasma membrane was completely disrupted (Figure 3C,D and Figure 4B,E, white arrows), and we observed leakage of the cytoplasmic content (Figure 3D) into the extracellular space and membrane blebbing on the amoeba surface (Figure 3C, asterisks).

**Figure 5:**
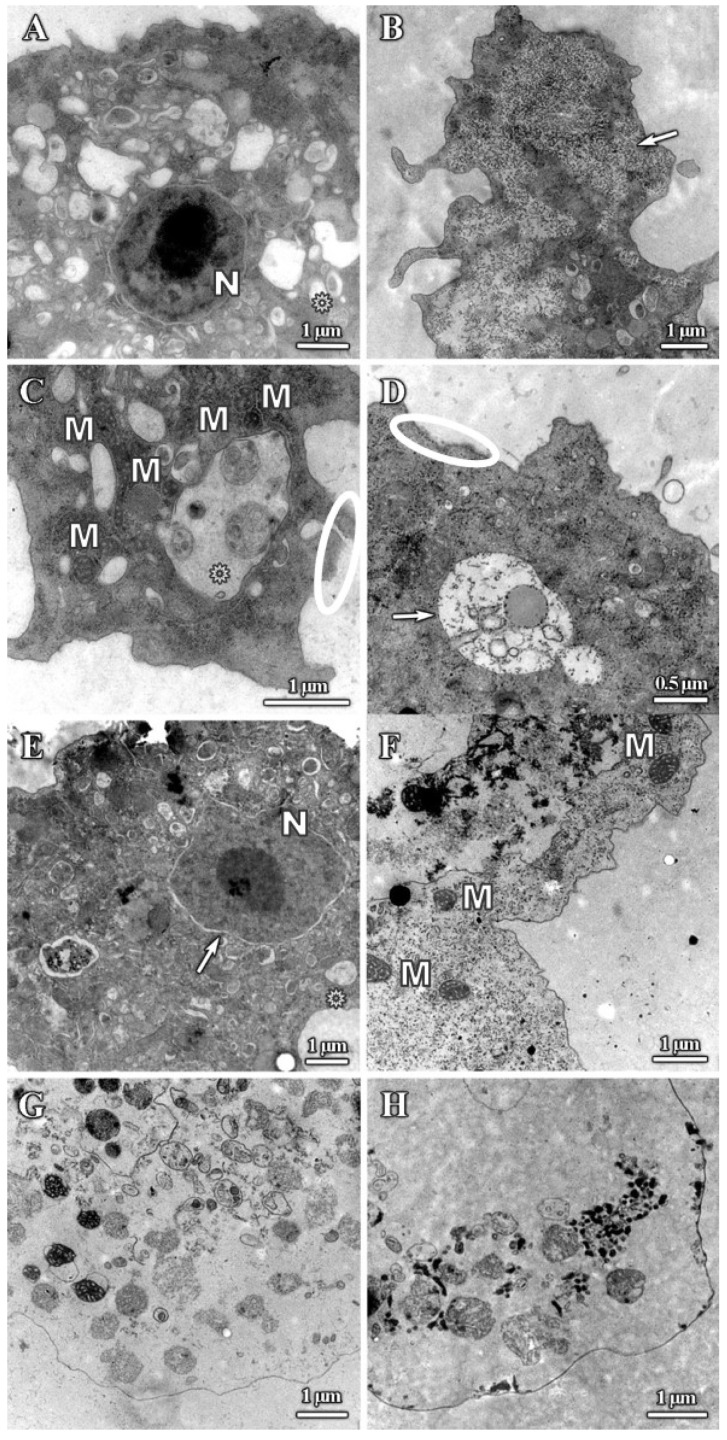
Transmission electron microscopy (TEM) micrographs of *A. polyphaga* trophozoites. Alterations observed after 24 h treatment. (**A**) 16 mg/L dendrimer **1**, (**B**) 16 mg/L dendrimer **1**, Arrow: granularity, (**C**) 16 mg/L dendrimer **1**, (**D**) 128 mg/L dendrimer **2**, (**E**) 64 mg/L dendrimer **3**, (**F**) 2 mg/L dendrimer **4**, (**G**) 2 mg/L dendrimer **4**, (**H**) 8 mg/L dendrimer **5**. Asterisk: Vacuoles. Circle: Plasma membrane damage. N: Nucleus. M: Mitochondria.

The results of the TEM analysis of *A. polyphaga* trophozoites are presented on Figure 5. The trophozoite ultrastructure was also altered compared with the control (Figure 2B), with treated trophozoites displaying a reduction in size and extensive plasma membrane damage (Figure 5C,D; circle). There was also a reduction in the number of acanthopodia, with trophozoite surfaces appearing smoother after all treatments (Figure 5A,B,D). In addition, dendrimer treatment caused an increase in the presence of cytoplasmic vacuoles (Figure 5A,C), an increase in cytoplasm granularity (Figure 5B arrow, Figure 5D,F), cytoplasmic disorganization (Figure 5A,B,D,F), and depletion of the cytoplasmic content after treating with the most effective compounds (Figure 5G,H). Treated trophozoites showed a greater number of mitochondria as untreated trophozoites (Figure 5C, dark structures) and lamellar bodies that might correspond with degradation process of mitochondria (Figure 5A,C; asterisks). Vacuoles with undefined content were also observed (Figure 5D, arrow), as were alterations of nuclei (Figure 5E, arrow) and aggregation of chromatin (Figure 5A). When trophozoites were extremely altered, the cytoplasm was completely disorganized, dense granules appeared, and organelles were deformed and no longer visible (Figure 5G,H).

### 3.2. Alterations on Cysts Morphology by SEM and TEM

**Figure 6:**
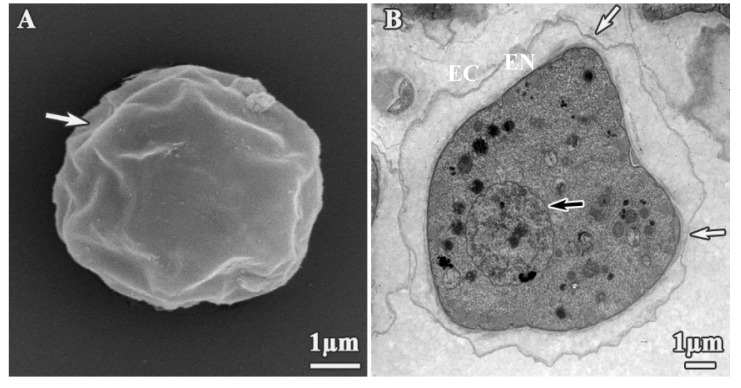
Untreated cysts of *A. polyphaga*. (**A**) SEM micrograph, (**B**) TEM micrograph. White arrows: Ostioles, black arrow: Nuclei and nucleoli. EC: Ectocyst. EN: Endocyst.

**Figure 7:**
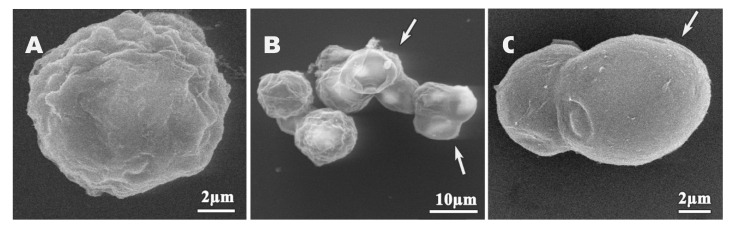
SEM micrographs of *A. polyphaga* cysts treated for 24 h. (**A**) 512 mg/L dendrimer **1**, (**B**) 128 mg/L dendrimer **4**, (**C**) 512 mg/L dendrimer **5**.

We next used SEM and TEM to assess structural and ultrastructural alterations of *A. polyphaga* cysts induced by dendrimer treatment. Untreated cysts exhibited the typical polygonal form (Figure 6A), with endocyst and plasma membrane clearly joined (Figure 6B). Ostioles (Figure 6A,B, white arrows) and a structured nucleus with the nucleoli were also clearly observed (Figure 6B, black arrow). Alterations to these features were observed upon dendrimer treatment, albeit at higher concentrations than those required to similar effects in trophozoites. These results were expected, and may be correlated with a reduction in the intake of dendrimers compounds due to nature of cyst walls. Polygonal shape was observed in some cysts (Figure 7A); however, the exocyst often appeared smoother (Figure 7B,C), while ostioles remained visible after treatments. In addition, cyst shrinkage was observed (Figure 7B, arrows) and perforations appeared on the cyst wall (Figure 7C, arrow).

**Figure 8:**
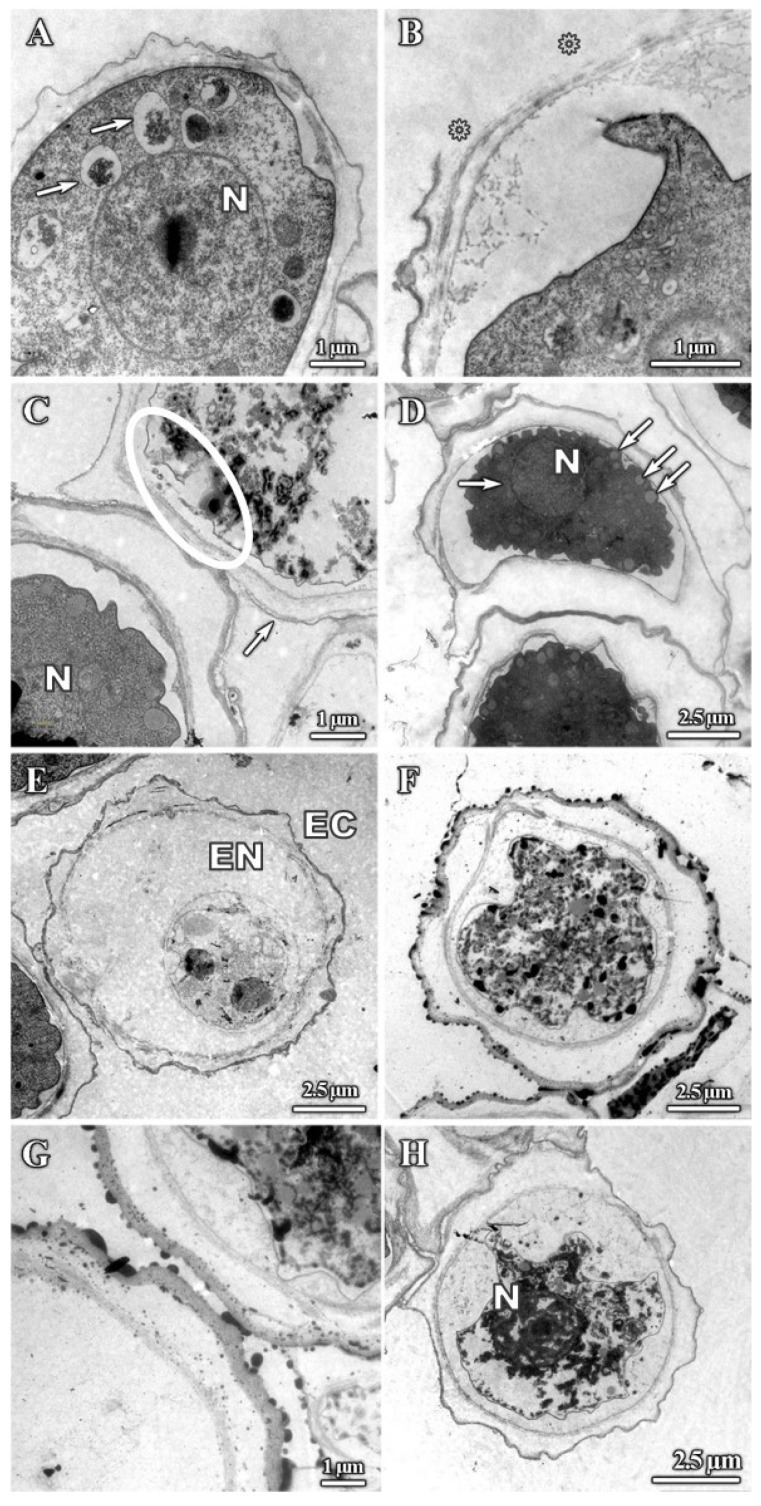
TEM micrographs of *A. polyphaga* cysts. Alterations in cysts after 24 h treatment. (**A**) 512 mg/L dendrimer **1**, (**B**) 512 mg/L dendrimer 1, (**C**) 512 mg/L dendrimer **1**. Circle: Plasma membrane alteration, (**D**) 512 mg/L dendrimer **2**, (**E**) 512 mg/L dendrimer **3**, (**F**) 128 mg/L dendrimer 4, (**G**) 128 mg/L dendrimer **4**, (**H**) 512 mg/L dendrimer **5**. N: Nucleus. EC: Ectocyst. EN: Endocyst.

**Figure 9:**
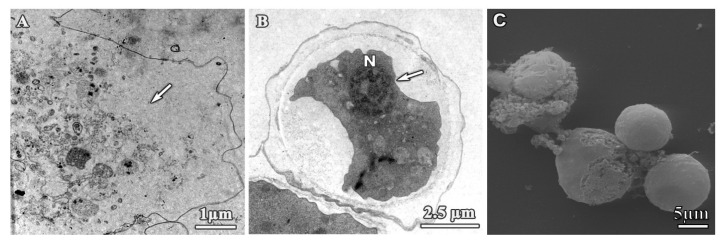
Micrographs of *A. polyphaga* incubated with digluconate chlorhexidine for 24 h. (**A**) TEM micrograph. Trophozoite, 2.5 mg/L. Arrow: Depletion of cytoplasmic content; (**B**) TEM micrograph. Cyst, 10 mg/L. Arrow: Cell shrinkage; (**C**) SEM micrograph. Trophozoites, 2.5 mg/L. N: Nucleus.

TEM imaging also revealed significant alterations to cyst ultrastructure. The majority of dendrimer-treated cysts showed intracystic amoeba shrinkage. This shrinkage resulted in the detachment of the plasma membrane from the endocyst (Figure 8A–H). Both the endocyst (Figure 8E) and plasma membrane (Figure 8G) were disrupted in some cases. The cyst wall was disrupted when treated with dendrimer 1 for 24 h (Figure 8B, asterisks); however, these effects were relatively infrequent, suggesting that this structure is largely resistant to dendrimer treatment. Other noticeable effects included the complete loss of cytoplasm integrity (Figure 8E–H) and the appearance of vesicles with unknown content (Figure 8A,D, arrows) and dense granules (Figure 8C,F,H). The absence of cytoplasmic contents suggests that they were leaked through the damaged plasma membrane (Figure 8C, circled area). Lastly, nuclei were also altered (Figure 8D) and aggregation of nuclear chromatin was observed (Figure 8H). Similar alterations were observed after treating trophozoites and cysts with chlorhexidine (Figure 9).

## 4. Discussion

The current treatment for *Acanthamoeba* keratitis is based on cationic antiseptics such as biguanides (Chlorhexidine or PHMB) in combination with a diamidine (propamidine isethionate) [23]. Cationic antiseptics lyse cells by binding to the phospholipid bilayer and causing fatal membrane damage [24], and diamidines interfere with methyl groups or directly disturbing amoebic nucleic acids [25]. The treatment is arduous, lengthy, and can be toxic to human corneal cells due to concentrations used and treatment duration, and resistance to these compounds has been reported [26]. Thus, there is a great need to identify new therapeutic agents against these protozoa.

Dendrimers are highly branched synthetic polymers with a wide range of applications due to their unique characteristics. They have been tested as antibacterial [14,19,27,28,29,30] or antiamoebic agents [16,17], as nanocarriers against bacteria, viruses, and parasites [31,32,33,34], and as nanocarriers for the delivery of nucleic acids [35], among others applications. The dendrimers used in these studies present two different cores: one core containing a silicon atom (Si) from which four branches extend, and the other containing a polyphenoxo core (P) attached to three branches. The nature of the core induces differences in the flexibility of the dendrimers, with compounds containing P cores being more flexible than those containing Si core, as the latter has a higher number of branches around the core. With respect to the dendrimer size, it has been reported that high generation dendrimers do not always correlate with the highest activity [36]. For this reason, dendrimers from generations 1 to 2 were tested in this work, as they had antiamoebic activity and were noncytotoxic against human and mammalian cell lines. Finally, we have tested dendrimers with two different terminal ammonium groups, –NH^3+^ and –NMe^3+^, to test the influence on the activity of these compounds on the ultrastructure of trophozoites and cysts. Dendrimer 4 showed the highest activity, with an IC_50_ of 2.4 + 0.1 mg/L. This is a first-generation dendrimer with a P core and six-NH^3+^ terminal groups on the periphery. As previously described, its activity levels might be due to the lesser substitution in the ammonium groups of its structure that leaves the amines free for further interactions with other molecules or structures [21].

We have evaluated the effect of cationic carbosilane dendrimers on trophozoites and cysts of a pathogenic strain of *A. polyphaga* isolated from a case of AK. Both SEM and TEM studies provided us with useful information about the potential mechanism of action of these compounds. Firstly, the trophozoites became rounded and acanthopodia disappeared. These structures, acanthopodia, are important for cellular adhesion, movements, and capturing food particles. It is likely that these structures play a role in the pathogenesis of *Acanthamoeba* because a binding to the corneal epithelium of the human host is necessary to establish infection [8]. Therefore, due to the ability of these cationic dendrimer molecules to reduce the number of acanthopodia it may be a useful additive to the composition of contact lens solutions as it may prevent trophozoite adhesion to the corneal epithelial cells. These molecules could be also useful in the treatment of AK, as it has been demonstrated that they exhibit amoebicidal effects against trophozoites and cysts at concentrations that are well tolerated by the human host cells [16,21].

We have shown other alterations caused by these dendrimers in vitro against trophozoites and cysts of *A. polyphaga*. The results showed effects on the plasma membrane, cell size reduction, loss of organelles, and cytoplasmic leakage. The alterations observed by our group are comparable to the ones observed by other researchers using different drugs [8,10,12]. When trophozoites were extremely altered, the organelles were destroyed, and the cytoplasm content was no longer visible. This phenotype has been previously described as “cells that looked like ghosts” [12], and similar observations in yeast were described as “empty bubbles” [37]. Lamellar bodies were observed in treated trophozoites, which might correspond with degradation process of mitochondria [38]. Our ultrastructural observations showed that dendrimers lead to membrane alteration and cytoplasmic leakage, likely resulting in the lysis of trophozoites or destruction of cyst wall. As the plasma membrane is necessary for cell integrity once the continuity of the membrane is damaged, trophozoites and cysts became osmotically fragile.

The aim of our study was to evaluate the morphological and structural alterations produced by different dendrimers on *A. polyphaga* trophozoites and cysts to provide insight into their possible mechanism of action. Interestingly, the phenotypes we observed following dendrimer treatment are consistent with the alterations caused by chlorhexidine [10]. The proposed mechanism of action of chlorhexidine (CLX) in *Acanthamoeba* has been suggested to be the same as the process described in bacteria [10,39,40,41]. Cationic dendrimers may have a similar mode of action because they are similarly positively charged [42]. Although the exact mechanism is not clear, these results provided us with evidence that the membranes are a clear target for these cationic dendrimers, which may interact with the negatively charged plasma membrane. As result of this interaction, cell permeability may be altered and membrane disruption may trigger cell rupture, affecting the integrity of both trophozoites and cysts. Consequently, these alterations may end in membrane disruption and cell death. Dendrimers may also penetrate inside cells and affect other organelle membranes, proteins, or DNA, disrupting cellular processes that are vital for *Acanthamoeba* survival. Further in vitro studies will be conducted using dendrimers conjugated to a fluorophore to study dendrimer internalization and the kinetics of the onset of the phenotypes we observed, as well as additional studies to confirm their efficacy in vivo. Together, these studies will provide further clarification as to whether these dendrimers may be considered as promising new therapeutic options for *Acanthamoeba* control and for *Acanthamoeba* keratitis infection treatment.

## 5. Conclusions

Our findings provided evidence that a clear target of these dendrimers is the plasma membrane, and showed that cytoplasmic damage also contributed to trophozoite and cyst death. Additionally, we observed fewer alterations on cysts that may be due to a reduced intake of the dendrimers due to nature of the cyst wall. Collectively, our results support that these compounds target membranes and their action was critical for parasite integrity.

## Figures and Tables

**Table 1:** Characteristics of dendrimers used.

Compound	R	Core	N	Functional Group	IC_50_ (mg/L)	MCC (mg/L)
[G_1_O_3_(S-NMe_3_)]^6+^	1	P	6	-NMe_3_^+^	16.9 + 0.8	>512
[G_1_Si(S-NMe_3_)]^4+^	2	S	4	-NMe_3_^+^	430.1 + 5.8	>512
[G_2_Si(S-NMe_3_)]^8+^	3	S	8	-NMe_3_^+^	46.5 + 0.9	>512
[G_1_O_3_(S-NH_3_)_6_]^6+^	4	P	6	-NH_3_^+^	2.4 + 0.1	256
[G_1_Si(NMe_3_)]^4+^	5	S	4	-NMe_3_^+^	7.8 + 0.2	512

**R**: Compound reference in text, **P**: Polyphenoxo, **S**: Silicon atom, **N**: Number of cationic peripheral functional groups, **IC_50_**: Inhibitory concentration 50, **MCC**: Minimum cysticidal concentration.
